# Prevention of low back pain: effect, cost-effectiveness, and cost-utility of maintenance care – study protocol for a randomized clinical trial

**DOI:** 10.1186/1745-6215-15-102

**Published:** 2014-04-02

**Authors:** Andreas Eklund, Iben Axén, Alice Kongsted, Malin Lohela-Karlsson, Charlotte Leboeuf-Yde, Irene Jensen

**Affiliations:** 1 Institute of Environmental Medicine, Unit of Intervention and Implementation Research, Karolinska Institutet, Nobels v13, S-171 77 Stockholm, Sweden; 2 Research Department, Spinecenter of Southern Denmark, Hospital Lillebælt, Østre Hougvej 55, DK-5500 Middelfart, Denmark; 3 Nordic Institute of Chiropractic and Clinical Biomechanics, Clinical Locomotion Network, Forskerparken 10A, 5230 Odense M, Denmark

**Keywords:** Chiropractic, Low back pain, Maintenance care, Manual therapy, Prevention, Randomized controlled trial, Secondary prevention, Spinal manipulation, Tertiary prevention

## Abstract

**Background:**

Low back pain (LBP) is a prevalent condition and a socioeconomic problem in many countries. Due to its recurrent nature, the prevention of further episodes (secondary prevention), seems logical. Furthermore, when the condition is persistent, the minimization of symptoms and prevention of deterioration (tertiary prevention), is equally important. Research has largely focused on treatment methods for symptomatic episodes, and little is known about preventive treatment strategies.

**Methods/Design:**

This study protocol describes a randomized controlled clinical trial in a multicenter setting investigating the effect and cost-effectiveness of preventive manual care (chiropractic maintenance care) in a population of patients with recurrent or persistent LBP.

Four hundred consecutive study subjects with recurrent or persistent LBP will be recruited from chiropractic clinics in Sweden. The primary outcome is the number of days with bothersome pain over 12 months. Secondary measures are self-rated health (EQ-5D), function (the Roland Morris Disability Questionnaire), psychological profile (the Multidimensional Pain Inventory), pain intensity (the Numeric Rating Scale), and work absence.

The primary utility measure of the study is quality-adjusted life years and will be calculated using the EQ-5D questionnaire. Direct medical costs as well as indirect costs will be considered.

Subjects are randomly allocated into two treatment arms: 1) Symptom-guided treatment (patient controlled), receiving care when patients feel a need. 2) Preventive treatment (clinician controlled), receiving care on a regular basis. Eligibility screening takes place in two phases: first, when assessing the primary inclusion/exclusion criteria, and then to only include fast responders, i.e., subjects who respond well to initial treatment. Data are collected at baseline and at follow-up as well as weekly, using SMS text messages.

**Discussion:**

This study investigates a manual strategy (chiropractic maintenance care) for recurrent and persistent LBP and aims to answer questions regarding the effect and cost-effectiveness of this preventive approach. Strict inclusion criteria should ensure a suitable target group and the use of frequent data collection should provide an accurate outcome measurement. The study utilizes normal clinical procedures, which should aid the transferability of the results.

**Trial registration:**

Clinical trials.gov; NCT01539863, February 22, 2012. The first patient was randomized into the study on April 13th 2012.

## Background

Low back pain (LBP) is a major public health problem in many countries with resulting consequences both for the individual and society. The total cost of LBP in Sweden was estimated at €1,840 million in 2001 [1]. Interventions that reduce the prevalence of back pain would therefore contribute to improvements on a public health level and potentially have large economic benefits.

LBP has been shown to be a recurrent and sometimes persistent phenomenon [2,3]. Logically, a recurrent and sometimes persistent condition that is highly prevalent invites the idea of prevention. However, knowledge about secondary prevention, to decrease the rate of recurrence, and tertiary prevention, to decrease the intensity or extent of persistent pain for this condition is scarce [4].

In Sweden, LBP is the condition for which most people seek care from a chiropractor [5,6]. Manual treatment, which has been shown to be effective for some patients [4], is a major treatment component. Among chiropractors, the traditionally employed long-term approach for individuals with recurrent or persistent pain is a preventive protocol described as maintenance care (MC) [7-10]. Early authors have described MC as “*…a regimen designed to provide for the patient’s continued well-being or for maintaining the optimum state of health while minimizing recurrences of the clinical status*” [11] and “*…treatment, either scheduled or elective, which occurred after optimum recorded benefit was reached, provided there was no evidence of relapse*” [12].

A 2008 systematic review concluded that chiropractic MC lacked an evidence-based definition as well as evidence-based indications, frequency, and content of treatment [13]. However, since 2009, several studies have investigated the frequency and content of this preventive strategy. As suggested by the name, the aim of the treatment is to maintain a certain treatment effect (i.e., less pain or improved function) [7], and care is normally delivered at widely spaced but regular intervals over a fairly long time [14,15].

Research during 2009 and 2010 has also concluded that there seems to be a general management concept within the chiropractic profession in the Scandinavian countries with regards to secondary and tertiary prevention [7,9,10,16]. In Sweden, 98% of chiropractors who have an academic education use some form of MC [7]. This type of care is in part proactive, i.e., the clinician will encourage the patient to perform specific exercises or a training program. Typically, one will also attempt to influence the LBP problem in other ways through discussions about life style [14]. Thus, regular visits may have a supportive psychosocial function by aiding coping strategies and providing support and guidance. Manual therapy (such as spinal manipulation or mobilization) of the musculoskeletal system is included as needed, as judged by the clinician [14,17,18].

A few studies have been published on the effect of preventive spinal manipulation on spinal pain and the results have been equivocal [5,19,20], maybe due to the fact that these were small studies that did not employ the evidence regarding indications for care in their inclusion process, nor regarding frequency and treatment content in their instructions for care. Thus, more research is needed to determine the effect and cost-effectiveness of this preventive package of care. In particular, it would be important to select study subjects in accordance with the procedures that have developed from years of professional experience and to include the treatment programs and activities that chiropractors actually use for this type of therapeutic strategy. This study makes use of the available evidence in the field.

## Methods/Design

### Study aim

The study aims to investigate the effect, cost-effectiveness, and cost-utility of preventive manual care as compared to manual care given only when there is a subject-perceived need in a population of patients with recurrent or persistent LBP. The primary outcome is the number of days with bothersome pain on which the cost-effectiveness calculations will also be performed. Thus, the null hypothesis will be that the subjects in the two treatment arms will report equal numbers of days with bothersome pain.

### Setting

Forty Swedish chiropractors will collect data on consecutive patients with recurrent or persistent LBP. The clinicians recruited for this study have participated in a previous study regarding the use of preventive care [8] and have experience of how to integrate research into clinical practice. Only clinicians who reported that they use preventive care selectively were recruited. This was thought to minimize the clinicians’ bias towards either of the treatment models. One two-hour training session, describing the project in detail, was given to the clinicians in a small group seminar format. Following the seminar, a member of the research team has been conducting weekly follow-ups by telephone with the participating clinicians to ensure that the procedures are followed according to the project outline and that the data collection is performed correctly.

### Eligibility criteria

The inclusion criteria for study subjects are: presenting with LBP as the main complaint, recurrent LBP (the individual has experienced episodes of LBP in the past), or persistent LBP (more than 30 days over the past year [21]). A minimum of three months must have passed since the last treatment by a chiropractor if previously treated. This interval was chosen as research indicates that the treatment seems to have little effect after three months [22]. This was also consistent with the research team’s clinical experience. The subjects must be of working age (18 to 65 years), have access to a mobile phone and knowledge of how to receive and send text-messages (SMS), as well as be proficient in Swedish. Subjects must rate themselves as “definitely improved” by the fourth visit [23]. The exclusion criteria for patients are: pregnancy, acute trauma, cancer, infection, cauda equina, osteoporosis, and vertebral fractures. Further, subjects with completely subsidized treatment from a third party payer, such as injury compensation [24], local county council subsidization, or workplace benefits, are excluded from the study. The majority of the chiropractic patients in Sweden do not have access to subsidized treatment.

An overview of the eligibility criteria can be found in Table 1.

**Table 1:** Eligibility screening

**Inclusion criteria**		**Exclusion criteria**
Baseline 1	Age 18 to 65 years	Pregnancy
	LBP with or without leg pain for more than 30 days during the past year	Chiropractic treatment less than 3 months ago
	Previous episodes	Completely subsidized treatment from third party payer
	Access to a mobile phone	Serious pathology (i.e., acute trauma, cancer, infection, cauda equina, osteoporosis, vertebral fractures) or contraindications to manual therapy
	Ability to send and receive SMS (text messages)	
Baseline 2	Self-rated “definitely improved” by the fourth treatment	Self-rated improvement being anything but “definitely improved” by the fourth treatment
Study start	Interval between treatments is one month or more	Interval between treatments never extends to one month

*LBP*, Non-specific low back pain.

### Procedures

**Figure 1:**
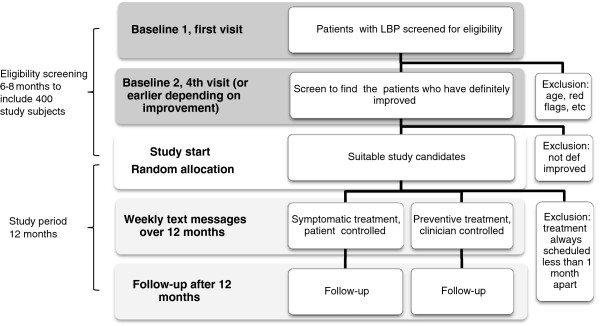
Study procedures.

The study procedures are shown in the flowchart in Figure 1. To resemble the clinical decision-making process, the eligibility procedure consists of two main stages; first, to find possible candidates and second, to select only the study subjects who respond favorably to treatment. According to previous research, this is congruent with the procedure used by chiropractors when making clinical decisions regarding MC [7,10].

For the initial visit, patients are instructed to arrive 15 minutes early if perceived as possible candidates for the study (i.e., presenting with LBP) when the booking is made. Upon arrival before the initial visit, the patients fill in a questionnaire, baseline 1, along with a screening form with inclusion/exclusion criteria with descriptive data. If no exclusion criteria exist, the patients are then treated as usual up until the fourth visit.

A continuous assessment regarding the response to treatment is made at each visit and recorded in the patients’ files. If the patients consider themselves “definitely improved” according to the global perceived improvement scale (single question with answer options definitely worse, probably worse, unchanged, probably better, definitely better) by the fourth visit or earlier, they are candidates for the study. During the fourth visit, or prior if “definitely improved”, the questionnaires of baseline 2 are administered.

The patients continue with treatment until the clinician considers them well enough to extend the interval between treatments to one month or longer. Clinically, the study subjects then enter the MC phase and data collection can commence, i.e., the study starts. The potential subjects are informed of the study procedure and asked to sign a letter of informed consent should they wish to participate. Subjects are then randomly allocated into one of two treatment arms; symptom guided treatment (patient controlled) or preventive/MC treatment (clinician controlled).

If patients do not improve to the point where the interval between treatments can be extended to at least one month, they are not eligible to continue in the study and are excluded. The cut-off interval of one month was decided after discussions within the research team based on their collective clinical experience. A time interval of less than one month was considered an active treatment regimen; this is also supported by a 2010 study [15].

Research suggests that the typical interval between treatments in an MC-regimen is two months [14]. However, a systematic review concluded that it was difficult to evaluate the effect of treatments more than three months apart [22]. The participating clinicians are therefore allowed to choose an interval between treatments of a minimum of one and a maximum of three months apart.

During the study period, study subjects in the two treatment arms continue treatment according to each protocol. The study period lasts 12 months. The treatments during these 12 months are offered with a reduced treatment fee of 50% for subjects in both treatment arms, but only for visits regarding LBP.

### Randomization procedure

Consecutively numbered opaque envelopes containing a letter with instructions are created off-site at the research center by a statistician and a research assistant. A 1:1 allocation ratio in randomly permuted blocks of different sizes according to a randomization schedule is used. SPSS v20 was used to generate the randomization code. The envelopes are opened consecutively in the presence of the study subjects as they become eligible for the study and give consent to participate.

### Blinding

The randomization is blinded to the study team in order not to influence the clinicians during the workshops and in personal contact during the inclusion process. The data collecting clinicians are unaware of the individual allocation of their patient until they have opened the envelope after consent is given. Due to the treatment modality, study subjects and clinicians cannot be blinded to the treatment.

### Sample size

A power analysis has estimated the number of subjects needed to detect a change in treatment effect of 30% (i.e., 30% fewer days of bothersome LBP) to be 177 in each treatment arm. The 30% cut-off point was decided to be a clinically important difference with regards to number of days of bothersome pain. Furthermore, a 20% (for acute pain) and 30% (for chronic pain) change in intensity measured on NRS-11 has been shown to be a clinically meaningful change [25,26].

In previous Swedish intervention studies among this group of chiropractors, compliance has been around 70 to 80% [27,28]. With a similar compliance rate in this study, we would need to include 400 study subjects when allowing for dropouts.

### Treatment arms

Each clinician is instructed to describe the two different treatment approaches as similar procedures, clinically both in use, when informing potential study subjects. The clinicians are asked to describe the aim of the study as a sincere wish to investigate if any of the two treatment modalities is more effective.

#### *Symptom guided treatment (patient controlled)*

Study subjects in the first treatment arm receive treatment according to their perceived needs, i.e., they are advised to seek care when the LBP returns, gets worse, or starts to affect their functional capacity. When seeking care, they get treated according to the clinicians’ judgments. As soon as the subjects are symptom-free or satisfied with the improvement, the treatment is discontinued, but can recommence should new symptoms appear. Thus, subjects in this group are therefore treated in relation to their self-reported symptoms and this treatment arm is described as “patient controlled”.

#### *Preventive treatment (clinician controlled)*

Study subjects in the second treatment arm receive preventive treatment according to the clinicians’ judgments, i.e., they are instructed to follow a treatment plan designed to minimize the recurrence of the pain/dysfunction or to maintain the effect of the initial treatment. In this group, the clinicians’ responsibility is to be proactive and to plan a preventive treatment strategy. This group is described as the “clinician controlled” treatment arm or the MC group. Visits are scheduled at 1 to 3 month intervals according to the clinicians’ judgments. If the subjects relapse before the next scheduled visit, they are instructed to seek care and are treated according to the clinical picture until they are symptom-free or satisfied with the improvement, after which they return to the original MC plan. The total number of treatments will be the sum of scheduled and acute visits.

For all study subjects, treatment content is decided by the treating clinician and the two arms will experience the same “individualized” care with regards to modalities and recommendations. Treatment content is noted in detail in the patients’ files.

### Outcome measures

The primary outcome of the study is the number of days with bothersome pain over 12 months*.* The term bothersomeness has previously been used as a measurement for the impact of pain [29-31]. The term has been shown to correlate well with self-rated health [32], pain intensity [33], disability, psychological health (anxiety, depression), and prediction of future work absence/healthcare consultations [34], and has been suggested as a standard outcome measure in LBP research [31].

During the follow-up time of 12 months, all participants receive weekly text messages by the SMS-Track system [35,36] asking “*How many days during the previous week has your low back pain been bothersome (i.e., affected your daily activities or routines)? Please answer with a number between 0 and 7*” (requiring an answer between 0–7, sent in a reply text message). This question has not been validated against other instruments; however, it has been used in previous studies [2,27,37] in similar settings in Sweden and Denmark and has been found useful for collecting data to examine the clinical course of LBP in the primary care sector. Should the subjects fail to respond, a subsequent SMS is sent after 48 hours with a reminder. If the participants fail to respond to this second SMS, a research assistant will follow-up with a phone call to investigate the reason for non-compliance and to provide further instructions if necessary.

Secondary measures consist of self-rated health (EQ-5D [38,39], a translated (Swedish) and validated questionnaire with five domains and three answer options in each), activity limitation (the Roland Morris Disability Questionnaire, RMDQ [40], a translated (Swedish) and validated questionnaire with 24 items requiring a yes/no response), pain intensity (the Numeric Rating Scale, NRS-11 [41,42]), psychological/behavioral characteristics (the West Haven-Yale Multidimensional Pain Inventory (MPI), [43-45], a translated (Swedish) and validated 34-item, 8 scales inventory divided into two parts), self-reported work absence [46] (single question, translated (Swedish) and validated) (Table 2), and work productivity (single question, based upon the validated but not translated WPAI-instrument (LBP–V2-Swedish) [47]) (Table 2).

**Table 2:** Assessment instruments and procedures

**Time**	**Instruments**	**Detailed description**
**First visit/baseline 1**	EQ-5D	Self-rated health [38]
	MPI-S	Psychological profile [43-45]
	NRS-11	Pain intensity last 24 hours [41,42]
	Descriptive data (subject)	Pregnancy (yes/no)
		Year of birth
		Pain in low back (yes/no)
		Pain in leg (yes, thigh only/yes, thigh and shin/no*)*
		Previously visited chiropractor (no/yes, more than 3 months ago/yes, less than 3 months ago*)*
		Past episodes of LBP (yes/no)
		Days in total with LBP during past year (<30/≥30)
		Pain in cervical or thoracic spine (no/yes, ≥30 days past year/yes, <30 days past year)
		Access to mobile phone (yes/no)
		Ability to send SMS text messages (yes/no)
		Belief in chance of improvement by treatment (NRS-11 varying from no chance to very probable)
		Subjective perception of health in general (5-step scale, perfect, very good, good, fair, poor)
**Baseline 2, fourth visit or earlier if indicated**	NRS-11	Pain intensity last 24 hours [41,42]
	Descriptive data (subject)	Subjective perception of health in general (5-step scale, perfect, very good, good, fair, poor)
	Descriptive data (clinician)	Clinician’s expectations concerning the response to preventive treatment
**Study start (When next visit can be scheduled at least one month ahead)**	RMDQ	Self-rated disability [40]
	Descriptive data (subject)	Sex (man/woman)
		Year of birth
		Profession (physically heavy labor/interchanging between heavy and light/ standing and walking/sitting)
	Self-reported work absence	Sick leave during past year (no/yes, 1–7 days in total/yes, 8–14 days in total/yes, ≥15 days in total)
	Descriptive data (clinician)	Type of treatment so far (manipulation, mobilization, activator, drop/soft tissue treatment/information, recommendations/other, as described by the chiropractor)
		Number of treatments during the past episode
**Random allocation**		
**Study period**	Weekly data collection for 52 weeks collected with SMS (text messages)	“How many days during the previous week has your low back pain been bothersome (i.e., affected your daily activities or routines)? Please answer with a number between 0 and 7” (requiring an answer between 0–7, sent in a reply text message [35,36])
**Follow-up after 12 months**	EQ-5D	Self-rated health [38]
	RMDQ	Self-rated disability [40]
	NRS-11	Pain intensity last 24 hours [41,42]
	Descriptive data (subject)	Other treatment in the past year? If yes, which type (physiotherapist, other chiropractor, medical doctor, medication, other)?/No
		Treatment value: considering economy, time consumption, LBP, function, quality of life, is the treatment worth continuing with? (5-step scale, definitely worth it, possibly worth it, equivocal, hardly worth it, definitely not worth it)
		Subjective perception of health in general (5-step scale, perfect, very good, good, fair, poor)
	Self-reported work absence	Sick leave during past year (no/yes, 1–7 days in total/yes, 8–14 days in total/yes, ≥15 days in total)
	Modified WPAI-LBP	Work productivity (How much has your low back pain affected your productivity during the past month, while at work? NRS-11 varying from LBP did not affect my work to LBP completely prevented me from working)
	Descriptive data (clinician)	Number of treatments including date
		Type of treatment so far (manipulation, mobilization, activator, drop/soft tissue treatment/information, recommendations/other, as described by the chiropractor)
		Reported side-effects (local soreness, fatigue, new radiating pain, other) including duration

*EQ-5D*, EuroQol 5 Dimensions; *LBP*, Non-specific low back pain; *MPI-S*, Multidimensional pain inventory, Swedish version; *NRS-11*, Numerical rating scale (11 steps, 0–10); *RMDQ*, Roland Morris Disability Questionnaire; *WPAI-GH*, Work productivity and activity impairment questionnaire – general health.

The secondary outcomes will be analyzed and reported independently of the primary outcome.

The primary utility measure of the study is quality-adjusted life years [38], and will be calculated using the EQ-5D questionnaire.

### Baseline and follow-up assessments

Baseline data at the initial visit (baseline 1) include demographic data (Table 2), pain intensity over the last 24 hours (NRS-11) [41,42], self-rated health (EQ-5D) [38], and the assessment of psychological profiles with the Multidimensional Pain Inventory, Swedish version (MPI-S) [43]. MPI-S will be used in a secondary analysis to investigate the potential interaction between the MPI subgroup and treatment effect.

During the fourth visit (or earlier if pain-free or well enough to discontinue care) (baseline 2), the subjects’ globally perceived improvement is measured using a 5-step scale (definitely worse, probably worse, unchanged, probably better, definitely better) [23] along with a follow-up NRS-11 [41,42] (for pain intensity over the past 24 hours). To assess clinicians’ expectations, the clinicians also state if they believe that their patient’s problem would benefit from preventive treatment (yes/no question).

Further baseline data are collected at the time of randomization. At that time, data regarding activity limitation (RMDQ [40]), physical work load, self-reported work absence [46], treatment content, and number of treatments are collected.

At the end of the study, both clinicians and study subjects receive a follow-up questionnaire (Table 2). The clinicians are asked to review the clinical files for treatment frequency, content, and any side-effects or adverse reactions associated with the treatment. The study subjects are asked about their pain intensity [41,42], activity limitations [40], general health [48], work absence [46] and satisfaction with care. To describe work productivity, a modified work productivity and activity impairment questionnaire, WPAI-instrument (LBP–V2-Swedish) [47] (How much has the pain affected productivity during past working month?) is used.

### Time line

The inclusion of study subjects started in April 2012 and is expected to proceed for a 24-month period due to the extensive inclusion protocol. The study *per se* runs over the course of 12 months and data collection is expected to be concluded during April 2015.

### Analysis

#### *Data analysis of the primary outcome “number of days with bothersome LBP”*

Intention-to-treat [49] will be used to test the null hypothesis as described in the study aim. Outcome measures will not be imputed for participants not responding to follow-up questions as previous research using the SMS-Track system has yielded high response rates [33,36,37] and research has indicated it unnecessary when performing analysis on longitudinal data [50]. Patient related outcomes measured at the fourth visit, weekly during the study period, and at the end of the study at 12 months will be evaluated using general [51] or generalized linear models [52-54] with mean baseline values as covariates. Sex, age, the presence of leg pain, patient expectations, pain intensity, the use of painkillers, sickness, albescence, type of profession, and number of treatments before being included in the study will be considered as possible confounders. All available data will be used for data analysis. A dropout analysis will be performed to compare the study population with dropouts regarding descriptive data. Additional analyses of secondary outcomes will be performed in an explorative manner.

An analysis of cost-effectiveness and cost-utility will be performed with consideration to both the individual and societal perspectives [55]. The cost-effectiveness will be determined by comparing the incremental cost effectiveness ratio in regard to the primary effectiveness measure between the two study arms [55]. Both the incremental cost effectiveness ratio and the incremental cost-utility ratio will be calculated by dividing the difference in mean total costs by the difference in the outcome of interest between the two treatment arms. Direct medical costs considered in the study are treatment cost (treatment fee) as well as time loss during treatment and travel (an average time for travel is estimated and added to an average patient visit time). Indirect costs such as production loss will be estimated using the human capital approach where lost time is valued using the hourly wage [56]. Since the data collection is performed between 2011 and 2014, all costs will be adjusted for inflation using a 3 to 5% inflation rate with the baseline year of the study as a base year [55] and calculated in €.

### Ethical aspects

The study will be conducted according to the guidelines of the Helsinki declaration [57] and good clinical research practice [58]. The project has been approved by the local ethical research committee at the Karolinska Institutet: 2007/1458-31/4. There is a risk that patients in the patient controlled arm could experience relapses that could have been prevented if an experienced clinician had decided on preventive treatment. Further, it is possible that the clinician-guided arm may have a negative impact on patients’ illness perceptions or empowerment. However, neither of the treatment arms differs from what the study subjects would have received should they not have participated in the study.

## Discussion

Although LBP is and has been a challenge for society for many years, little is known about the preventive strategies available. Within the chiropractic profession there is a management culture with a preventive intent towards recurrent and persistent LBP. For the individual patient, the possibility of preventing episodes may be very beneficial and help keep the individual active. Furthermore, the ability to control symptoms and their effects on everyday life may be paramount. However, a preventive treatment with potential side effects and related costs can only be justified if it is demonstrated to have clinically worthwhile effects.

To date, there has been no large scale randomized clinical trial comparing chiropractic MC for LBP with symptom-guided treatment. This study will utilize available evidence in the field for the selection of study subjects, frequency of treatment, and treatment content. Furthermore, the study mimics the usual clinical procedures of chiropractic care, which will aid the transferability of the study results.

### Trial status

The trial is ongoing and patients are being recruited. Patient recruitment started in April 2012 and is expected to continue until April 2014.

## Abbreviations

EQ-5D: EuroQol 5 Dimensions; LBP: Non-specific low back pain; MC: Maintenance care; MPI-S: Multidimensional pain inventory; NRS-11: Numerical rating scale (11 steps, 0–10); RMDQ: Roland Morris Disability Questionnaire; WPAI-GH: Work productivity and activity impairment questionnaire – general health.

## Competing interests

None of the authors have any competing interests to disclose. None of the funding bodies are involved in or have influence over the design of the study, the interpretation of the results, or the decision to publish the results.

## Authors’ contributions

AE is the main author, drafted the manuscript and has been involved in project management. IA has been involved in supervision and project management. AK has been consulted in matters of study design. MLK has served as an expert in health economics issues. CLY has served as an expert with regards to epidemiological and design issues. IJ has been involved in project management and supervision as well as contributing as an expert in epidemiological and design issues. All authors have been involved in the planning and design of the study as well as critical revision and intellectual improvement of the manuscript. All authors have read and approved the final manuscript.

## Authors’ information

AE is a part-time clinically active chiropractor in a private practice and is a PhD student at the Karolinska Institutet funded by the Institute for Chiropractic and Neuro-musculoskeletal Research and the European Chiropractors’ Union. IA is a part-time clinically active chiropractor in a private practice and has a post-doctoral position at the University of Southern Denmark funded by the Danish Chiropractic Research Foundation. AK has a research position at the University of Southern Denmark funded by the Danish Chiropractic Research Foundation. MLK has a post-doctoral position at the Karolinska Institutet. CLY has a research position at the University of Southern Denmark funded by the Lillebaelt Hospital and the Danish Chiropractic Research Foundation. IJ is the head of the Unit of Intervention and Implementation Research at the Karolinska Institutet.
